# Advanced disease and CD8^+^ TEMRA cells predict severe infections in multiple myeloma

**DOI:** 10.3389/fimmu.2025.1532645

**Published:** 2025-02-12

**Authors:** Eva Tranter, David Busch, Clarissa Heck, Igor Wolfgang Blau, Axel Nogai, Phillip Schiele, Christian Meisel, Lars Bullinger, Marco Frentsch, Il-Kang Na

**Affiliations:** ^1^ Charité – Universitätsmedizin Berlin, corporate member of Freie Universität Berlin, Humboldt-Universität zu Berlin, Medizinische Klinik m. S. Hämatologie, Onkologie und Tumorimmunologie, Berlin, Germany; ^2^ Onkologische Schwerpunktpraxis Tiergarten, Berlin, Germany; ^3^ Berlin Institute of Health (BIH) at Charité Universitätsmedizin Berlin, BIH Center for Regenerative Therapies (BCRT), Berlin, Germany; ^4^ Institute of Medical Immunology, Charité-Universitätsmedizin Berlin, Berlin, Germany; ^5^ Department of Immunology, Labor Berlin-Charité Vivantes GmbH, Berlin, Germany; ^6^ German Cancer Consortium (DKTK) Partner Site Berlin, German Cancer Research Center (DKFZ), Heidelberg, Germany; ^7^ ECRC Experimental and Clinical Research Center, Charité - Universitätsmedizin Berlin, corporate member of Freie Universität Berlin and Humboldt Universität zu Berlin, Berlin, Germany

**Keywords:** Multiple Myeloma, MGUS, infection, risk score, secondary immunodeficiency

## Abstract

**Introduction:**

Infections are a major cause of early morbidity and mortality in patients with multiple myeloma (MM) who are characterized by immunodeficiency secondary to disease. However, prospectively collected data on infection risk in this population are scarce. We aimed at identifying parameters in monoclonal gammopathy of undetermined significance (MGUS) and newly diagnosed MM (NDMM) patients with predictive power for early severe infections (SI).

**Methods:**

We conducted a prospective study with newly diagnosed MGUS and NDMM patients. Besides clinical and laboratory data, immune parameters were collected at initial diagnosis before therapy initiation. Primary endpoint was the occurrence of SI within 12 months after diagnosis.

**Results:**

45% of patients developed infection, 26% with SI. Four main risk factors for SI were identified: ECOG ≥ 2 (p < 0.001), ISS stage II/III (p = 0.002), therapeutic intervention (p < 0.001), and elevated CD8^+^ TEMRA cells (p = 0.027). A risk score was compiled, enabling the stratification of patients with low or high risk for SI with a sensitivity of 92.9% and a specificity of 80%.

**Conclusion:**

We developed a straightforward risk score that considers the relevance of T cell fitness in MGUS and NDMM patients and can help physicians to identify patients at risk of infection, thus enabling the implementation of timely and individualized prevention strategies.

## 1 Introduction

Improving the prediction of infections and establishing stronger correlations between laboratory markers and clinically relevant endpoints has been identified as a critical need by an international expert panel, emphasizing the importance of developing novel biomarkers (1). Monoclonal gammopathy of undetermined significance (MGUS) is a highly prevalent precancerous state in adults above 50 years of age, characterized by monoclonal plasma cell proliferation in the bone marrow and potential progression to multiple myeloma MM (2). The impairment of the immune system due to the underlying disease, particularly the humoral deficiency that accompanies both the diagnoses of MGUS and MM, plays a crucial role. It has been shown that MM patients, and to a lesser extent MGUS patients, exhibit significantly lower antibody titers against common pathogens compared to age-matched controls (3), which explains the increased risk of infections with encapsulated pathogens such as *haemophilus influenzae* and *streptococcus pneumoniae*. Additional immune abnormalities beyond hypogammaglobulinemia have been detected in MM patients (4, 5). Some of these findings have been associated with clinical manifestations: it has been suggested that patients who would develop infections early after initial diagnosis exhibited lower numbers of circulating CD19^+^ B-cells compared to those who remained infection free. Further, high CD19^+^ B-cell numbers have been associated with a decreased incidence, severity, and mortality from infections and with better overall survival (6, 7). Notably, antineoplastic therapy is also known to exacerbate pre-existing SID (4, 5, 8–10).

Several routine clinical and laboratory parameters, particularly those denoting aggressive disease, have been identified as predictors for infection risk, including international staging system (ISS) stage, low hemoglobin, low platelet count, high β2-microglobulin (β2-MG), elevated lactate dehydrogenase (LDH) and serum calcium levels (11–16).

Significant advances in therapeutic strategies in recent years have transformed MM into a chronic disease. Nevertheless, the early mortality rate of MM patients remains high (17). In addition to deaths due to progressive disease, heart and kidney failure, infections are one of the leading causes of early mortality, accounting for approximately 40% of early deaths in MM (18).

Here, we provide data from a prospective single-center trial analyzing clinical and laboratory characteristics of MGUS and newly diagnosed MM (NDMM) patients and propose an easy-to-use prognostic tool to identify patients at increased risk of early severe infections (SI). To the best of our knowledge, this is the first study combining a broad range of immune parameters with clinical features and routine laboratory tests in the prognostic analysis, thus capturing and ranking the impact of disease-related immune dysfunctions on infectious complications in MGUS and NDMM.

Our aim was to identify parameters in MGUS and NDMM patients that have a reliable predictive power for SI within the first year after diagnosis to facilitate the timely implementation of individualized prevention strategies.

## 2 Methods

### 2.1 Patients

64 patients with MGUS or NDMM who presented to our outpatient or inpatient center between 01/2019 – 09/2022 were prospectively enrolled. Clinical as well as an array of immunological parameters (**Supplementary Table 1**) and laboratory data (**Supplementary Table 2**) were investigated at time of study inclusion, in any case before the initiation of a specific therapy, including corticosteroids. Patients were monitored for infectious complications within the first year after initial diagnosis. All patients gave their informed consent, and the study was approved by the Ethics Committee of Charité-Universitätsmedizin Berlin.

### 2.2 Classifications

Infections were graded according to the Common Terminology Criteria for Adverse Events (CTCAE) v5.0; SI were classified as infections of CTCAE grade 3 or higher. In case of multiple SI, the most severe was considered for the analysis. Early SI were defined as severe infections occurring within one year after initial diagnosis. Clinical performance status of patients was categorized using the ECOG performance status (19). All NDMM patients were classified using the ISS from 2005 (20).

### 2.3 Experimental analyses

Enumeration and phenotyping of naïve and memory T-cell subsets was performed in EDTA whole blood samples using accredited test methods (DIN EN ISO 15189) at the Department of Immunology at Labor Berlin, as described previously (21). Briefly, the following mouse anti-human fluorescently-labelled monoclonal antibodies (all from Beckman Coulter, Krefeld, Germany) were used: CD3 APC-A750 (clone UCHT1), CD4 ECD (clone SCFI12T4D11), CD8 APC (clone B9.11), CD45RA Pacific-Blue (clone J33), and CCR7 PE (clone G043H7). Stained samples were acquired on a ten-color Navios EX flow cytometer and analyzed using Navios Software (Beckman Coulter). For gating strategy see **Supplementary Figure 1**. Interleukin-8 was measured from patients’ plasma after lysis of erythrocytes by immunoassay using an Immulite 1000 (Siemens, München, Germany) and Immulite Kit LK8P1 (Siemens, München, Germany).

### 2.4 Statistical analysis

Patients were grouped according to the occurrence of SI. Group 1: with no or non-severe infections, group 2: with SI (CTCAE ≥ 3). Data points that were >1.5 times the interquartile range below quartile one or >1.5 times the interquartile range above quartile three were considered outliers. The data set was cleaned from outliers by removing patients from the analysis with outliers in >50% of parameters (n = 3). Missing numerical values were imputed with the mean if absolute skewness was < 0.5, otherwise the median was used. Categorical features were imputed with the mode (highest frequency). Statistical significance of numerical variables was calculated using student’s t-test. Pearson’s correlation coefficients were used to calculate linear correlations among all significant numerical features for internal validation and cross-correlation. Statistical significance of categorical variables was calculated using chi-squared (χ^2^) test. P-values <0.05 were defined as statistically significant. 26 numerical variables met statistical significance threshold and were retained for expert review (**Supplementary Table 1**). Of these, clinically significant numerical parameters were selected and transformed into discrete variables by setting cut-offs according to laboratory reference values or, if not applicable, clinically feasible cut-off values. Statistically significant variables were selected by expert assessment based on their clinical and/or biological relevance to be included in a multiple logistic regression model. In addition, results of the correlation analysis were included in parameter selection. The final model included four variables. A scoring system was developed in which factors were assigned scores based on their coefficient in the multivariate logistic model. Based on the cumulative score, patients were categorized into groups with high (≥7 points) or low (<7 points) risk of severe infection.

## 3 Results

### 3.1 Patient characteristics

A total of 64 patients were enrolled in our analysis; seven patients were excluded due to loss of follow up or non-newly diagnosed disease; three patients were excluded during statistical analysis due to outliers in >50% of investigated parameters. Characteristics of analyzed patients are summarized in **Table 1**. We analyzed a total of 54 patients, 16 diagnosed with MGUS and 38 with NDMM irrespective of treatment intention (watch and wait, non-intensive or intensive therapy). Median age was 66 years (SD ± 13.5) in the MGUS cohort and 60 years (± 11.2) in the NDMM with even distribution by sex (27 males, 27 females). Most patients exhibited an ECOG 0-1, only 15.8% showed an ECOG of 2 or higher (**Table 1**). Concerning prognostic grading of NDMM, 65.8% of patients were categorized as ISS I, 13.2% as ISS II, and 21% as ISS III. In the NDMM cohort, 31.6% received no therapy; 39.5% of patients received a high-intensity therapy including autologous stem cell transplantation (autoTx) while 28.9% of patients received low-intensity treatment (**Table 1**). All NDMM patients received continuous *pneumocystis jirovecii* prophylaxis with sulfamethoxazole/trimethoprim as well as acyclovir in a kidney-function adjusted, prophylactic dose. Patients who underwent autoTx received ciprofloxacin 500 mg bidaily during neutropenia until reaching leukocytes >1/nl, continuous acyclovir prophylaxis in a kidney-function adjusted dose as well as *pneumocystis jirovecii* prophylaxis with sulfamethoxazole/trimethoprim after sufficient engraftment.

**Table 1:** Patient characteristics.

	NDMM (n = 38)	MGUS (n = 16)
Age (years, ± SD)	60 (± 11.2)	66 (± 13.5)
Gender		
Female Male	17 (44.7%) 21 (55.3%)	10 (62.5%) 6 (37.5%)
Comorbidities		
Chronic kidney disease Cardiovascular disease Diabetes mellitus	4 (10.5%) 5 (13.2%) 6 (15.8%)	2 (12.5%) 1 (6.3%) 2 (12.5%)
ECOG		
0 1 2 3 4	22 (57.9%) 10 (26.3%) 5 (13.2%) 1 (2.6%) 0	13 (81.25%) 3 (18.75%) 0 0 0
ISS		
I II III	25 (65.8%) 5 (13.2%) 8 (21%)	n/a
Therapeutic intervention		
no therapy Non-intense therapy including Anti-CD38+ mAB Intense therapy incl. autoTx including Anti-CD38+ mAB	17 (44.7%) 7 (18.4%) 4 (10.5%) 14 (36.8%) 5 (13.2%)	n/a
IVIG		
Yes No Unknown	4 (10.5%) 32 (84.2%) 2 (5.3%)	2 (12.5%) 13 (81.25%) 1 (6.25%)
Infections (CTCAE grade)		
0-2 3 4 5	25 (65.8%) 7 (18.4%) 5 (13.2%) 1 (2.6%)	15 (93.7%) 1 (6.3%) 0 0

n/a = not applicable.

### 3.2 Characteristics of early SI

24 of 54 patients developed an infection within the first year of initial diagnosis, 14 patients experienced infections classified as severe (CTCAE 3 or higher) (**Table 1**). In these 14 patients, we documented a total of 20 infections during the observation period. SI occurred almost exclusively in the group of NDMM patients. Prevalence of early SI increased with advancing ISS stage. The majority of SI occurred before or during induction therapy (60% of SI). In most cases, no pathogen was identified. However, a bacterial infection was found in around one third of SI patients and a viral infection in 10% of SI patients. Most SI were respiratory tract infections (40.9%). Detailed information on all SI can be found in **Table 2**. Though study recruitment was in part conducted during the SARS-CoV-2 pandemic, we only observed one severe (CTCAE grade 3) SARS-CoV-2 infection in our cohort.

**Table 2:** Infection characteristics.

	CTCAE ≥3 (n=20)*
Pathogen detected	
bacterial viral SARS-CoV-2 unknown	7 (35%) 2 (10%) 1 (5%) 11 (55%)
Infection site^α^	
respiratory FUO blood stream bowel urinary tract central nervous system endocarditis joint infection	9 (40.9%) 4 (18.2) 2 (9.1%) 2 (9.1%) 2 (9.1%) 1 (4.5%) 1 (4.5%) 1 (4.5%)
Time of occurrence	
before therapy during induction therapy during autoTx after autoTx/during maintenance phase	5 (25%) 7 (35%) 2 (10%) 6 (30%)

*14 patients developed 20 severe infections. All severe infections are considered here.

^α^all infectious sites noted; in some cases more than one infection site was noted during the same course.

### 3.3 Clinical characteristics and biomarkers associated with early SI

**Figure 1:**
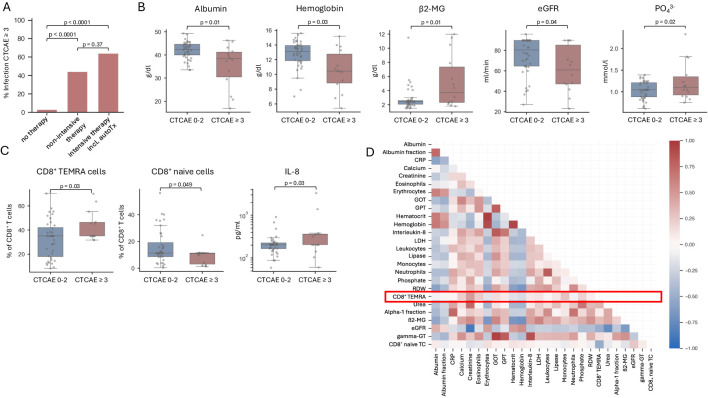
Risk factors associated with early severe infection in MGUS and NDMM patients. **(A)** Bar graph illustrating the percentage of severe infections (CTCAE 3 or higher) in MGUS and NDMM patients receiving no therapy, non-intensive therapy or intensive therapy including autologous stem cell transplantation within the first year after initial diagnosis. n=54. **(B, C)** Box plots showing parameters denoting aggressive disease and T cell exhaustion are associated with the occurrence of severe infections (CTCAE 3 or higher). Significance was calculated using a student’s t-test. n=54. **(D)** The figure depicts a correlation matrix of relevant laboratory parameters analyzed using Pearson’s correlation coefficient. The inclusion of parameters was based on their statistical significance, determined through prior analysis using a student’s t-test. n=54.

We aimed at identifying relevant parameters associated with SI occurring within the first year after diagnosis. We found 26 parameters to be significantly associated with the occurrence of SI (**Table 3**). The following parameters were selected for further evaluation due to clinical significance: albumin, hemoglobin, β2-MG, LDH, serum calcium, urea, creatinine, eGFR, inorganic phosphate, absolute neutrophil count, the alpha1-fraction of serum immunofixation, and CD8^+^ terminally differentiated memory T-cells [CD3^+^/CD8^+^/CD45RA^+^/CCR7^-^, which are often referred to as CD45RA+ effector-memory T-cells (TEMRA)]. For these laboratory parameters, clinically feasible and analytically implementable cut-off values were set (**Table 4**). In the univariate analysis, we identified NDMM diagnosis compared to MGUS (p < 0.001), ISS stage II and III (p = 0.002), ECOG ≥ 2 (p < 0.001), and therapeutic intervention (p < 0.001) as highly significant clinical parameters. The commencement of therapy was in any case associated with the occurrence of early SI, this effect was even more pronounced, when an intensive therapy regimen containing autoTx was administered (**Figure 1A**). Nine patients (23.7%) received a therapy containing an anti-CD38 antibody such as Isatuximab or Daratumumab. Four of these nine patients developed an SI, in the group receiving conventional therapy, ten out of 16 patients developed an SI. We found no significant differences in the occurrence of infections depending on the use of anti-CD38 antibody therapy (data not shown). Age, sex, and presence of comorbidities were not associated with the occurrence of early SI (**Table 4**). Key lab findings included albumin <35 g/l, anemia with hemoglobin <10 g/l, elevated β2-MG >5.5 mg/l, reduced estimated glomerular filtration rate (eGFR) <60 ml/min/1,73 m², and elevated serum phosphate (PO_4_^3-^) >1.45 mmol/l, all tied to a higher SI risk (**Table 4**, **Figure 1B**). A comprehensive summary of all investigated parameters and their corresponding p-values, calculated using Student’s t-test, is provided in **Supplementary Table 3**.

**Table 3:** All laboratory parameters significantly associated with occurrence of severe infections in MGUS and MM patients as determined by student’s t-test.

Parameter	P-Value
**Albumin**	**0.00046**
Hematocrit	0.00058
**Hemoglobin**	**0.00120**
Erythrocytes	0.00120
Glutamic oxaloacetic transaminase (GOT)	0.00184
Red cell distribution width (RDW)	0.00235
**β2-microglobulin**	**0.00259**
Lipase	0.00271
**Urea**	**0.00614**
Albumin fraction (serum immune fixation)	0.00695
Eosinophiles, absolute	0.00733
**Lactate dehydrogenase (LDH)**	**0.01023**
Creatinine	0.01044
**Neutrophils, absolute count**	**0.01131**
Gamma-Glutamyltransferase (y-GT)	0.01354
**Alpha1 fraction (serum immune fixation)**	**0.01574**
C-reactive protein (CRP)	0.01770
Monocytes, absolute	0.02087
**Anorganic phosphate (PO^4^_3-_)**	**0.02475**
**CD8^+^ TEMRA cells (CD3^+^/CD8^+^/CD45RA^+^/CCR7^-^)**	**0.02608**
Leukocytes, absolute	0.02635
Interleukin-8	0.03347
**eGFR**	**0.03843**
**Calcium**	**0.04469**
**Naïve CD8^+^ T-cells (CD3^+^/CD8^+^/CD45RA^+^/CCR7^+^)**	0.04952
GPT	0.04985

The parameters highlighted in bold were transformed into categorical variables for further analysis by selecting clinically/biologically meaningful cut-offs.

**Table 4:** Univariate analysis of influencing factors in patients with and without severe infections.

Factor	Investigated cases (%) (n = 54)	Group with infections CTCAE ≥3 (%) (n = 14)	Group with infections CTCAE < 3/no infections (%) (n = 40)	χ2 P-value
Sex male female	27 (50%) 27 (50%)	10 (71%) 4 (29%)	17 (42%) 23 (57%)	0.120
Age < 65 years 65-79 years ≥ 80 years	31 (57%) 19 (35%) 4 (7%)	7 (50%) 7 (50%) 0	24 (60%) 12 (30%) 4 (10%)	0.252
Diagnosis MGUS MM	16 (30%) 38 (70%)	1 (7%) 13 (93%)	15 (37%) 25 (63%)	**< 0.0001**
ISS^**x**^ I II III	16 (30%) 25 (46%) 13 (24%)	1 (7%) 5 (36%) 8 (57%)	15 (38%) 20 (50%) 5 (12%)	**0.002**
ECOG 0-1 ≥ 2	48 (89%) 6 (11%)	8 (57%) 6 (43%)	40 (100%) 0 (0%)	**< 0.0001**
Therapy yes intensive incl. autoTx non-intensive no	23 (43%) *14 (26%)* *9 (17%)* 31 (57%)	13 (93%) *9 (64%)* *4 (29%)* 1 (7%)	10 (25%) *5 (12.5%)* *5 (12.5%)* 30 (75%)	**< 0.0001** **<0.0001** **<0.0001**
Comorbidity* yes no	19 (35%) 35 (65%)	6 (43%) 8 (57%)	13 (33%) 27 (67%)	0.709
Albumin (g/l) < 35 ≥ 35	7 13%) 47 (87%)	5 (36%) 9 (64%)	2 (5%) 38 (95%)	**0.013**
Hemoglobin (g/dl) < 10 ≥ 10	8 (15%) 46 (85%)	5 (36%) 9 (64%)	3 (8%) 37 (92%)	**0.034**
β2-mg (mg/l) < 5.5 ≥ 5.5	47 (87%) 7 (13%)	9 (64%) 5 (36%)	38 (95%) 2 (5%)	**0.013**
LDH (U/l) < 225 > 225	29 (54%) 25 (46%)	6 (43%) 8 (57%)	23 (57%) 17 (42%)	0.526
Calcium (mmol/l) < 2.5 ≥ 2.5	43 (80%) 11 (20%)	9 (64%) 5 (36%)	34 (85%) 6 (15%)	0.204
Urea (mg/dl) < 48 ≥ 48	44 (81%) 10 (19%)	9 (64%) 5 (36%)	35 (88%) 5 (12%)	0.127
Creatinine (mg/dl) < 1.5 ≥ 1.5	47 (87%) 7 (13%)	10 (71%) 4 (29%)	37 (92%) 3 (8%)	0.119
eGFR (ml/min/1,73 m²) < 60 ≥ 60	14 (26%) 40 (74%)	7 (50%) 7 (50%)	7 (18%) 33 (82%)	**0.042**
PO_4_^3-^ (mmol/l) < 1.45 ≥ 1.45	51 (94%) 3 (6%)	11 (79%) 3 (21%)	40 (100%) 0 (0%)	**0.019**
α1-fraction < 4.5 ≥ 4.5	43 (80%) 11 (20%)	10 (71%) 4 (29%)	33 (82%) 7 (18%)	0.617
Neutrophils (/nl) < 4.5 ≥ 4.5	41 (76%) 13 (24%)	8 (57%) 6 (43%)	33 (82%) 7 (18%)	0.127
TEMRA cells in % of CD8^+^ T-cells ≤25 > 25	14 (26%) 40 (74%)	0 (0%) 14 (100%)	14 (35%) 26 (65%)	**0.027**

x Patients with MGUS are not considered here.

*Comorbidities included: diabetes mellitus, chronic kidney disease, cardiovascular disease (coronary artery disease, congestive heart failure, severe valvular disease).

The bold letters describe the statistical significance.

### 3.4 Association of TEMRA cell levels with early SI in MGUS and NDMM patients

Interestingly, we found that patients with SI had higher CD8^+^ TEMRA cells (CD3^+^/CD8^+^/CD45RA^+^/CCR7^-^) relative to all CD8^+^ T-cells at time of first diagnosis as compared to those who did not develop early SI (**Figure 1C**). Furthermore, we observed a significant increase in the inflammatory-mediating chemokine IL-8 in patients with SI (**Figure 1C**). Of note, when investigating NDMM patients separately, we observed a strong trend towards higher CD8^+^ TEMRAs (p = 0.05017) as well as elevated IL-8 levels (p = 0.05036) in patients developing SI (**Supplementary Figure 2**). To further analyze the subgroup of patients with elevated CD8^+^ TEMRAs at initial diagnosis, we have analyzed potential differences between patients with low and high CD8^+^ TEMRAs concerning other laboratory parameters and clinical characteristics. Aside from the incidence of severe infections, no significant differences were observed. Interestingly, the shift towards high CD8^+^ TEMRAs led to an overall reduction in naïve, effector memory, and central memory T-cells. Data on additional T-cell and B-cell populations as well as cytokines are summarized in **Supplementary Figures 3**–**5** and **Supplementary Table 3**.

### 3.5 CD8^+^ TEMRA cells as an independent risk factor for SI in MGUS and MM patients

Using a correlation matrix of Pearson’s coefficients for all parameters significantly associated with the occurrence of SI, our analysis confirmed correlations between markers of advanced disease, such as those indicating impaired kidney function and hematopoiesis, along with β2-MG and LDH (**Figure 1D**). Intriguingly, CD8^+^ TEMRA cells and consequently CD8^+^ naïve T-cells did not demonstrate significant correlations with other parameters. Consequently, CD8^+^ TEMRA cells merit recognition as an independent risk factor for SI.

### 3.6 Individual risk prediction for SI in MGUS and NDMM patients by multiple logistic regression modeling

**Figure 2:**
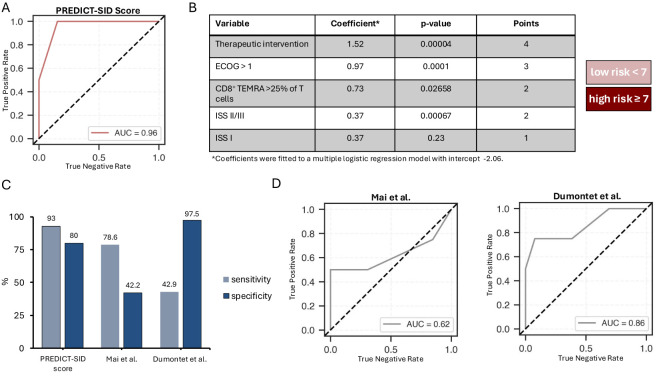
PREDICT score for the stratification of MGUS and NDMM patients at high risk of early severe infection. **(A)** Assessment of the presented multiple logistic regression model depicting the receiver operating characteristic (ROC) curve and the area under der ROC curve (AUC). **(B)** Summary of the variables included in the PREDICT risk score with presentation of their coefficients, p-values and the points assigned to the variables; patients with a score <7 are at low, patients with a score >= 7 are at high risk for severe infections (CTCAE 3 or higher. **(C)** Bar graph imaging the sensitivity and specificity of the presented risk score as well as two other published risk scores when applied to our dataset. **(D)** Assessment of the multiple logistic regression model considering the risk stratifiers proposed by Mai et al. and Dumontet et al., depicting the receiver operating characteristic (ROC) curve and the area under the curve (AUC).

To discern the risk of early SI in MGUS and NDMM patients, a logistic regression model was employed. Four key variables emerged as paramount predictors of early SI: therapeutic intervention, ISS stage, ECOG, and the relative abundance of CD8^+^ TEMRA cells. The model achieved an overall accuracy of 88%, with a recall of 75%, and an area under the curve (AUC) of 0.96 (**Figure 2A**). A risk score, titled PREDICT-SID (Prediction of **s**econdary immun**o**deficiency in MGUS and NDMM), was developed based on four primary risk factors identified in the study: induction of MM therapy, ECOG, ISS stage, and CD8^+^ TEMRAs. Each risk factor was converted into a point value according to the coefficient (**Figure 2B**). Additive scoring was used to classify patients into two risk groups: low (0-6 points) and high risk (≥7 points). The PREDICT-SID score shows a sensitivity of 92% and a specificity of 80%. Furthermore, we compared PREDICT-SID against two existing risk scores (15, 16), both based on retrospective analyses; characteristics are summarized in **Table 5**. First, we evaluated infection risk for each patient in our cohort using the published scores by Dumontet et al. (16) and Mai et al. (15), calculating an individual risk score based on each patient’s clinical and laboratory parameters according to each model. We then assessed the performance of each score in predicting SI by comparing the predicted risk classifications with the observed outcomes. This comparison allowed us to compute sensitivity and specificity via a confusion matrix: in our cohort, the Dumontet et al. score, which incorporates ECOG, β2-MG, hemoglobin, and LDH, yielded a sensitivity of 42.9% and specificity of 97.5%, while the Mai et al. score, which considers platelet count, ECOG, ISS, and age, achieved a sensitivity of 78.6% and specificity of 42.4% (**Figure 2C**). Secondly, the risk stratifiers from the two published scores were incorporated into a logistic regression analysis of our dataset, and the area under the receiver operating characteristic (ROC) curve was calculated for both scores, showing an inferior ROC and AUC of the two published scores (**Figure 2D**) when compared to PREDICT-SID (**Figure 2A**).

**Table 5:** Comparison of risk scores for the assessment of risk for severe infections MM and MGUS patients.

Study	Patient characteristics	Endpoint(s)	Risk stratifiers
This study	NDMM and MGUS patients	Infections CTCAE ≥3 within 1 year after diagnosis	ECOG, ISS, therapeutic intervention, CD8^+^ TEMRA cells
Mai et al., 2023 (15)	Transplant eligible, receiving induction	infections CTCAE ≥3, death, infections CTCAE ≥3 or death	Platelet count, ISS, ECOG, age
Dumontet et al., 2018 (16)	Transplant ineligible MM patients, receiving Rd/MPT induction	Treatment emerging infections CTCAE ≥3 in the first 4 months	ECOG, β2-MG, hemoglobin, LDH

## 4 Discussion

In this prospective observational study, we investigated the risk factors associated with early SI in MGUS and NDMM patients linking performance status, markers of advanced disease, therapeutic intervention, and TEMRA cells to an increased SI risk within the first year after diagnosis.

Our findings underscore the clinically imminent and substantial burden of infections in this patient population, with 46% of patients experiencing infections of any severity and over a quarter of patients developing SI (CTCAE ≥ 3) within the first year. These results align with previous studies showing that NDMM patients have an early infection risk ranging widely between 11 and 78% (11, 15, 16, 22), depending largely on the performance status and/or transplant eligibility of the analyzed patient cohort. The majority of infections occurred during the first 3-12 months following MM diagnosis (15, 17, 22–24), contributing significantly to early morbidity and mortality. MGUS patients, though typically less immunocompromised than NDMM, also face heightened infection risks due to humoral insufficiency (3, 25). For instance, a population-based study reported a twofold increased risk of bacterial and viral infections in MGUS patients, including significantly higher risks for pneumonia and septicemia (26). Furthermore, the elevated susceptibility to infection has been highlighted in studies of infection-related outcomes, such as increased morbidity and mortality following COVID-19 infection (27, 28). Recommendations published by the European Myeloma Network in 2014 also highlighted the increased risk of infections and the associated higher mortality in MGUS patients compared to the general population (29). These observations highlight the need for early risk stratification in MGUS and NDMM patients, allowing for individualized preventive measures to be implemented at diagnosis, potentially reducing the incidence of serious infections and improving patient outcomes in this vulnerable population.

In the presented study, markers of advanced disease, e.g. ISS stages II and III, low albumin and hemoglobin levels, elevated β2-MG, impaired renal function, and antineoplastic therapy were found to be strongly associated with SI. We did not find an increase in infections in patients being treated with anti-CD38 antibodies. However, the small number of patients receiving therapy including anti-CD38 antibodies must be acknowledged as a limitation. The influence of antineoplastic therapy on immune competence of MM patients has been manifoldly described (4, 5, 8–10, 30). In our study, the start of antineoplastic therapy was among the most significant contributors to the risk of SI. This effect was even more pronounced when intensive treatment including autoTx was performed. This underscores the delicate balance between disease control and immunosuppression in MM treatment, necessitating careful consideration of infection prevention strategies during treatment, especially during induction therapy.

Consistent with prior analyses, we confirmed a poor performance status, as measured by ECOG score, to be strongly associated with SI, emphasizing the impact of overall health and functional physical capacity on infection incidence in NDMM and MGUS patients (15, 16, 22).

Our study uniquely assessed immunological parameters’ influence on infection in MGUS and NDMM (**Supplementary Table 1**). Patients with SI exhibited increased CD8^+^ TEMRA cells and IL-8 levels compared to patients who do not suffer from infectious complications. TEMRA cells are antigen experienced and are usually considered to be terminally differentiated T-cells that may arise as a result of prolonged antigen exposure, such as that seen in chronic infection with cytomegalovirus or Epstein-Barr virus (31). TEMRA cells are characterized by low proliferative capacity as well as high sensitivity to apoptosis (32). It has been proposed that prolonged antigen exposure leads to a distortion in the TCR repertoire, evidenced by the oligoclonality observed in TEMRA cells (33), potentially resulting in compromised defense against new infections (34). CD8^+^ TEMRA cells have also been found to accumulate in the bone marrow of MM patients and were characterized to be functionally severely impaired, displaying features of exhaustion and senescence (35). The authors of the study hypothesized that this T-cell-mediated secondary immunodeficiency is driven by myeloma cells and can be partly cured by treating the underlying disease.

The observed higher abundance of TEMRA and fewer naïve CD8^+^ T-cells in patients developing early SI may reflect a reduced capability to recognize and adequately response to new pathogens, contributing to the increased susceptibility to infections, as has been reported elsewhere (34). The problematic higher frequencies of TEMRA cells in some patients can be also relevant in the context of modern therapeutic strategies, such as bispecific antibodies and CAR-T cell therapies, which rely on the functional competency of T-cells for efficacy (36, 37). Understanding the baseline immunological status, including TEMRA cells and more generally T-cell dysfunction, thus holds dual significance: it aids in identifying high-risk patients for early SI and might inform the stratification for T-cell engaging immunotherapies.

To translate our findings into clinical practice, we have created a straightforward risk score, PREDICT-SID, that can be used to identify MGUS and NDMM patients at high risk of early SI. By combining clinical patient and routine laboratory characteristics, and, for the first time, immunological parameters, we created a compact prediction model based on four parameters: ISS stage, ECOG, MM therapy, and CD8^+^ TEMRA cells.

Our model, unlike previously published scores (15, 16) that focused solely on advanced disease markers and performance, incorporates an immune dysregulation marker, achieving superior model performance with a sensitivity of 93%, a specificity of 80%, and an AUC of 0.96. The discrepancies in the results obtained by the scoring systems are most likely due to the underlying differences in the characteristics of the patients studied. Mai et al. only included patients who were eligible for autoTx, while Dumontet et al. included patients who were not eligible for transplantation and were therefore likely to be in poorer general health. In contrast, the present study included both patients eligible for transplantation and those not eligible for transplantation, as well as MGUS patients under a watch and wait strategy.

Despite the good performance of the model, our study is limited by its sample size and single-center design with a heterogeneous patient cohort including MGUS and NDMM patients, all with different treatment indications and eligibility. However, a notable strength of the present study lies in its prospective design and the assessment of immunologic characteristics including T-cell subset characterization and cytokine profiling, allowing the clinical relevance of these characteristics to be determined.

Our findings underscore the complexity of infection risk in MGUS and NDMM patients and highlight the need for a multifaceted approach to risk assessment. We were able to show that if patient risk factors e.g. poor performance status, advanced disease, and elevated TEMRA cell levels concur with the need for antineoplastic therapy, the risk of a potentially life-threatening infection rises significantly. Integrating risk stratification, like the PREDICT-SID score, into clinical care could aid in early intervention and tailored treatment strategies, reducing ‘overtreatment’. As we move forward, prospective validation of our model and further exploration of T-cell dysfunctions in MGUS and MM will be crucial in refining our understanding of infection risk dynamics and optimizing patient care.

## Data Availability

The raw data supporting the conclusions of this article will be made available by the authors, without undue reservation.
