# Supplementary material for: Advanced disease and CD8+ TEMRA cells predict severe infections in multiple myeloma
Source: Front Immunol. 2025 Feb 12;16:1532645. doi: 10.3389/fimmu.2025.1532645 (PMC11862831; doi:10.3389/fimmu.2025.1532645)
Supplement: Supplementary file 1 [file DataSheet1.docx]

Supplementary Material

# Supplementary Tables

## Supplementary Table 1. Immunological parameters investigated.

| Parameter | Method |
| --- | --- |
| IgG1 | immunoturbidimetry |
| IgG2 | immunoturbidimetry |
| IgG3 | immunoturbidimetry |
| IgG4 | immunoturbidimetry |
| Anti-Tetanustoxoid IgG | ELISA |
| Pneumococcus antibody aPCP IgGg | ELISA |
| Pneumococcus antibody aPCP IgG2 | ELISA |
| Granulocytes, absolute | flow cytometry |
| Granulocytes, relative | flow cytometry |
| Lymphocytes, absolute | flow cytometry |
| Lymphocytes, relative | flow cytometry |
| Monocytes, absolute | flow cytometry |
| Monocytes, relative | flow cytometry |
| NK cells, absolute | flow cytometry |
| NK cells, relative to lymphocytes | flow cytometry |
| CD19^+^ cells, absolute | flow cytometry |
| CD19^+^ cells, relative to lymphocytes | flow cytometry |
| CD3^+^ cells, absolute | flow cytometry |
| CD3^+^ cells, relative to lymphocytes | flow cytometry |
| CD4^+^ cells, absolute | flow cytometry |
| CD4^+^ cells, relative to lymphocytes | flow cytometry |
| CD4^+^ cells, relative to T-cells | flow cytometry |
| CD8^+^ cells, absolute | flow cytometry |
| CD8^+^ cells, relativ to lymphocytes | flow cytometry |
| CD8^+^ cells, relative to T-cells | flow cytometry |
| CD8^-^/CD4^-^ cells, relative to T-cells | flow cytometry |
| CD8^+^/CD4^+^ cells, relative to T-cells | flow cytometry |
| CD4^+^/CD8^+^ ratio | flow cytometry |
| Monocyte HLA-DR expression | flow cytometry |
| Naive CD45RA^+^/CCR7^+^, realtive to CD8^+^ cells | flow cytometry |
| TEMRA cells (CD45RA^+^/CCR7^-^, relative to CD8^+^ cells) | flow cytometry |
| Central memory cells (CD45RA^-^/CCR7^+^, relative to CD8^+^ cells) | flow cytometry |
| Effector memory cells (CD45RA^-^/CCR7^-^, relative to CD8^+^ cells) | flow cytometry |
| Naive CD45RA^+^/CCR7^+^, realtive to CD4^+^ cells | flow cytometry |
| TEMRA cells (CD45RA^+^/CCR7^-^, relative to CD4^+^ cells) | flow cytometry |
| Central memory cells (CD45RA^-^/CCR7^+^, relative to CD4^+^ cells) | flow cytometry |
| Effector memory cells (CD45RA^-^/CCR7-, relative to CD4^+^ cells) | flow cytometry |
| CD45RA^+^, relative to CD4^+^ cells | flow cytometry |
| CD45RA^-^, relative to CD4^+^ cells | flow cytometry |
| Treg cells (CD25^+^/CD127^-^), relative to CD4^+^ cells | flow cytometry |
| Naive Treg cells (CD45RA^+^), relative to CD4^+^ cells | flow cytometry |
| Effector Treg cells (CD45RA^-^), relative to CD4^+^ cells | flow cytometry |
| Treg cells, absolute | flow cytometry |
| Naive B-cells, relative | flow cytometry |
| Naive B-cells, absolute | flow cytometry |
| Marginal zone like B-cells, relative | flow cytometry |
| Marginal zone like B-cells, absolute | flow cytometry |
| lgM only memory B-cells, relative | flow cytometry |
| lgM only memory B-cells, absolute | flow cytometry |
| Switched memory B-cells, relative | flow cytometry |
| Switched memory B-cells, absolute | flow cytometry |
| Transitional B-cells, relative | flow cytometry |
| Transitional B-cells, absolute | flow cytometry |
| CD21^low^/CD38^low^ B-cells, relative | flow cytometry |
| CD21^low^/CD38^low^ B-cells, absolute | flow cytometry |
| Switched plasmablasts, relative | flow cytometry |
| Switched plasmablasts, absolute | flow cytometry |
| Soluable IL2-receptor | ELISA |
| IL-8 | ELISA |
| Mannose-binding lectin | ELISA |
| ConA-stimulated TNFα | cytokine bead array |
| ConA-stimulated INFγ | cytokine bead array |
| ConA-stimulated IL-2 | cytokine bead array |
| ConA-stimulated IL-4 | cytokine bead array |
| ConA-stimulated IL-5 | cytokine bead array |
| ConA-stimulated IL-10 | cytokine bead array |

## Supplementary Table 2. Routine laboratory parameters investigated.

| Parameter |
| --- |
| Sodium |
| Potassium |
| Calcium |
| Calcium, corr. |
| Anorganic PO4 |
| Magnesium |
| Chloride |
| Iron |
| Transferrin saturation |
| Ferritin |
| Creatinine |
| eGFR (CKD-EPl) |
| Urea |
| Uric acid |
| Bilirubin, total |
| Bilirubin, direct |
| Protein |
| Albumin |
| Serum electrophoresis (incl. albumin fraction, alpha1-globulin, alpha2-globulin, beta-globulin, gamma-globulin) |
| M-protein |
| Light chains, lambda |
| Light chains, kappa |
| f-kappa/f-lambda ratio |
| C-reactive protein |
| Haptoglobin |
| ß-2-microglobulin |
| Cholesterol, total |
| HDL cholesterol |
| non-HDL cholesterol |
| LDL cholesterol |
| Triglycerides |
| Glutamic-oxaloacetic transaminase (GOT) |
| Glutamic-pyruvic transaminase (GPT) |
| Amylase |
| y-Glutamyltranspeptidase (yGT) |
| Lipase |
| LDH |
| TSH |
| Hemoglobin |
| Hematocrit |
| Erythrocytes |
| Leukocytes |
| Thrombocytes |
| Mean corpuscular volume (MCV) |
| Mean corpuscular henoglobin (MCH) |
| Mean corpuscular hemoglobin concentration (MCHC) |
| Mean platelet volume (MPV) |
| Red cell doistribution width (RDW-CV) |
| Neutrophils, absolute |
| Immature Granulocates, absolute |
| Lymphocytes, absolute |
| Lymphocytes, relative |
| Monocytes, absolute |
| Eosinophils, absolute |
| Basophils, absolute |
| Retikulocytes, absolute |
| Retikulocytes, relative |
| Reti.production index |
| Reticulocyte Hemoglobin |
| Quick |
| INR |
| aPTT |
| HIV1/2-Ab ,P24-AG |
| HIV1/2-Ab ,P24-AG |
| HIV1/2-Ab ,P24-AG |
| Hep C virus antibody in serum |
| Hep C virus antibody in serum |
| Hep C virus antibody in serum |
| C3 complement |
| C4 complement |
| ACE |
| Creatinkinase (CK) |
| Alk.Phosphatase |
| Cytomegalovirus IgM-antibody (EIA) |
| Cytomegalovirus IgG-antibody (EIA) |
| EBV-IgM (EIA) |
| EBV-VCA-IgG (EIA) |
| EBV-EBNA1-IgG (EIA) |
| EBNA1-IgG |
| EBV-IgM (Imm.blot) |
| VCA-p23-IgM |
| EA-p138-IgM |
| EA-p54-IgM |
| EBV-IgG (Imm.blot) |
| VCA-p18-IgG |
| VCA-p23-IgG |
| EA-p138-IgG |
| EA-p54-IgG |
| IEA-BZLF1-IgG |
| IEA-ZEBRA-IgM |

## Supplementary Table 3. P-values for all investigated parameters.

P-values were determined using Student’s t-tests to compare patients who developed severe infections (CTCAE grade ≥3) with those who did not.

| PARAMETER | P-VALUE |
| --- | --- |
| Albumin | **0.00046** |
| Henatocrit | **0.00058** |
| Hemoglobin | **0.00120** |
| Erythrocytes | **0.00120** |
| GOT | **0.00184** |
| Red cell distribution width | **0.00235** |
| Beta2MG | **0.00259** |
| Lipase | **0.00271** |
| Urea | **0.00614** |
| Albumin fraction (serum immunofixation) | **0.00695** |
| Eosinophiles, absolute | **0.00733** |
| LDH | **0.01023** |
| Creatinine | **0.01044** |
| Gamma GT | **0.01354** |
| a1 fraction (serum immunofixation) | **0.01574** |
| C-reactive protein | **0.01770** |
| Inorganic phosphate | **0.02475** |
| IgA | **0.02545** |
| CD45RA+CCR7-TEMRA, % CD8+ | **0.02608** |
| Leukocytes, absolute | **0.02635** |
| IL-8 | **0.03347** |
| eGFR | **0.03843** |
| Calcium | **0.04469** |
| Naïve CD45RA+CCR7+, %CD8plus | **0.04952** |
| GPT | **0.04985** |
| Central memory CD45RA-CCR7+, %CD8+ | 0.05248 |
| CD45RA+CCR7-TEMRA, %CD4+ | 0.05921 |
| Beta-fraction (serum immunofixation) | 0.07103 |
| vitamin_D3 | 0.07392 |
| Immature Granulocytes, absolute | 0.08669 |
| Basophils, absolute | 0.08745 |
| Bilirubin, total | 0.08941 |
| CD8-CD4-, %T-cells | 0.09352 |
| Ferritin | 0.09506 |
| aPCP_IgG2 | 0.09540 |
| Immature Granulocytes, % | 0.09806 |
| aPCP IgGg | 0.10140 |
| HDL | 0.11195 |
| Lymphocytes, % | 0.13846 |
| Neutrophils,_absolute | 0.15612 |
| Monocytes, absolute | 0.16532 |
| Mannose binding Lectin | 0.17932 |
| IL-10 | 0.18297 |
| lgM-only-Memory-B-cells, % | 0.19489 |
| IL-4 | 0.20879 |
| Uric acid | 0.21089 |
| CD4/D8 ratio | 0.21712 |
| Granulocytes, % | 0.21843 |
| Triglyceride | 0.22011 |
| Central memory CD45RA-CCR7+, % CD4+ | 0.22226 |
| IgM | 0.26151 |
| IgG2 | 0.26262 |
| Switched memory B-cells, % | 0.27695 |
| Non-HDL | 0.29720 |
| Monocytes, % | 0.30472 |
| Protein, total | 0.30873 |
| M-Protein | 0.33509 |
| TNFalpha | 0.34473 |
| Treg, absolute | 0.34486 |
| Transitional_B-cells | 0.35980 |
| IgG4 | 0.38437 |
| Age | 0.38577 |
| CD4+, absolute | 0.40374 |
| Free lambda light chain | 0.40852 |
| Anti Tetanustoxoid IgG | 0.42132 |
| LDL | 0.44696 |
| CD45RA-, % CD4+ | 0.45352 |
| CD45RA+, % CD4+ | 0.45354 |
| CD8+CD4-, % T-cells | 0.45951 |
| naive_B-cells | 0.45964 |
| Transitional B-cells, absolute | 0.48658 |
| INFgamma | 0.49723 |
| CD4+, % Lymphocytes | 0.50903 |
| CD3+, absolute | 0.51275 |
| Thrombocytes | 0.51775 |
| Basophils, % | 0.52243 |
| IgE | 0.52869 |
| Switched memory B-cells, absolute | 0.54946 |
| a2 fraction (serum immofixation) | 0.55066 |
| Haptoglobin | 0.55499 |
| Marginal zone-like B-cells, absolute | 0.55976 |
| CD19+, absolute | 0.56072 |
| sIL2-R | 0.56255 |
| Sodium | 0.56309 |
| Switched plasmablasts, % | 0.56517 |
| CD21lowCD38Iow_B-cells, absolute | 0.56648 |
| Amylase | 0.56777 |
| Neutophils, % | 0.57121 |
| Cholesterol | 0.58566 |
| MPV | 0.60298 |
| CD8+, absolute | 0.60804 |
| lgM_only_Memory_B cells, absolute | 0.61478 |
| Switched plasmablast, absolute | 0.61535 |
| Free kappa light chains | 0.62276 |
| gamma_fraction (serum immunofixation) | 0.64347 |
| HbA1c (mmol/mol hemoglobin) | 0.64472 |
| HbA1c | 0.65127 |
| MCH | 0.65813 |
| Effector memory CD45RA-CCR7, %_CD8+ | 0.66160 |
| IgG3 | 0.66981 |
| Potassium | 0.67822 |
| CD8+, %_Lymphocytes | 0.68416 |
| Naïve Treg CD45RA+, % CD4+ | 0.68504 |
| Total protein (serum) | 0.68597 |
| IL-2 | 0.68863 |
| Monocyte HLA-DR expression | 0.71400 |
| Kappa/lambda ratio | 0.71718 |
| MCV | 0.72528 |
| IL-5 | 0.73266 |
| Naïve CD45RA+CCR7+, % CD4+ | 0.73273 |
| Effector_Treg_CD45RA-, %_CD4+ | 0.75116 |
| CD8+, % T-cells | 0.75671 |
| Transferrin saturation | 0.75875 |
| CD19+, % Lymphocytes | 0.75979 |
| Effector memory CD45RA-CCR7-, % CD4+ | 0.76467 |
| Marginal zone-like B-cells, % | 0.79373 |
| Iron | 0.80482 |
| NK-cells, % Lymphocytes | 0.81061 |
| Lymphocytes, absolute | 0.83201 |
| NK-cells, absolute | 0.86094 |
| Treg CD25+127-, % CD4+ | 0.86820 |
| Chloride | 0.87156 |
| naive_B-cells, absolute | 0.87273 |
| Eosinophils, % | 0.87494 |
| Monocytes, % | 0.87678 |
| IgG | 0.88567 |
| IgG1 | 0.90477 |
| CD21lowCD38Iow B-cells, % | 0.90999 |
| CD3+, % Lymphocytes | 0.92766 |
| MCHC | 0.94366 |
| Magnesium | 0.96205 |
| CD4+, % T-cells | 0.98341 |
| TSH | 0.99298 |

# Supplementary Figures

**Supplementary Figure 1.** **Gating strategy for T-cell subsets in human peripheral blood.** T-cell subsets were analyzed by flow cytometry as followed. Briefly, T-cells were identified using a region set on CD3+ leukocytes in a CD45 vs SSC dot plot showing all leukocytes (A). Within CD3+ T-cells, CD4+ and CD8+ single positive T-cells were identified in a CD4 vs. CD8 dot plot (B). Naïve and memory CD4+ or CD8+ T-cell subsets (C, D) were identified according to their differential expression of the CD45RA and CCR7 (naïve: CD45RA+ CCR7+; central memory: CD45RA−CCR7+; effector memory: CD45RA− CCR7−; terminally differentiated effector memory (TEMRA): CD45RA+ CCR7−).

Supplementary Figure 2. Figure 4. Subanalysis of NDMM patients. A: Bar graphs depicting distribution of non-severe (CTCAE 0-2) and severe infections (CTCAE 3-5) among NDMM patients with different ECOG performance status, ISS stage, therapeutic intervention and low albumin (<35g/l) vs. high albumin (≥ 35 g/l). n= 38. B-D: Box plots showing parameters denoting aggressive disease and T cell exhaustion are associated with the occurrence of severe infections (CTCAE 3 or higher). Significance was calculated using a student’s t-test. n= 38.

Supplementary Figure 3. Box plots showing association of T-cell subpopulations with the occurrence of severe infections (CTCAE 0-2 vs. CTCAE 3-5). Significance was calculated using a student’s t-test. n=54.

Supplementary Figure 4. Box plots showing association of B-cell subpopulations with the occurrence of severe infections (CTCAE 0-2 vs. CTCAE 3-5). Significance was calculated using a student’s t-test. n=54.

Supplementary Figure 5. Box plots showing association of cytokines with the occurrence of severe infections (CTCAE 0-2 vs. CTCAE 3-5). Significance was calculated using a student’s t-test. n=54.
